# Predictors and Drivers of End-of-Life Medicare Spending Among Older Adults with Solid Tumors: A Population-Based Study

**DOI:** 10.3390/cancers17061016

**Published:** 2025-03-18

**Authors:** Courtney E. Baird, Elizabeth Wulff-Burchfield, Pamela C. Egan, Lee A. Hugar, Ami Vyas, Nikolaos A. Trikalinos, Michael A. Liu, Adam J. Olszewski, Leonidas E. Bantis, Orestis A. Panagiotou, Emmanuelle Bélanger

**Affiliations:** 1 Center for Gerontology and Healthcare Research, Department of Health Services, Policy and Practice, Brown University School of Public Health, Providence, RI 02903, USA; 2 Medical Oncology Division and Palliative Medicine Division, Department of Internal Medicine, University of Kansas School of Medicine, University of Kansas Cancer Center, The University of Kansas Health System, Kansas City, KS 66160, USA; 3 Department of Medicine, The Warren Alpert Medical School of Brown University, Providence, RI 02903, USA; 4 Department of Genitourinary Oncology, H. Lee Moffitt Cancer Center, Tampa, FL 33612, USA; 5 Department of Pharmacy Practice, College of Pharmacy, University of Rhode Island, Kingston, RI 02881, USA; 6 Division of Oncology, Department of Medicine, Washington University School of Medicine, St. Louis, MO 63110, USA; 7 Siteman Cancer Center, St. Louis, MO 63110, USA; 8 Herbert Irving Comprehensive Cancer Center, Columbia University Medical Center, New York, NY 10032, USA; 9 Department of Biostatistics and Data Science, University of Kansas Medical Center, Kansas City, KS 66160, USA

**Keywords:** end-of-life care, palliative care, geriatric oncology, Medicare spending, cancer

## Abstract

**Purpose:** Medicare patients who die from cancer are responsible for about 30% of annual Medicare spending, most of which occurs during the last 30 days of life. Yet, there are significant and persisting knowledge gaps regarding which factors contribute to this high-intensity EoL spending. To that end, we conducted a retrospective analysis of SEER-Medicare data to identify risk factors associated with high-intensity EoL spending among older adults who died of breast, prostate, lung, or colorectal cancer. **Methods:** We used multivariable linear regression to identify clinical, demographic, socioeconomic, and geographic characteristics associated with the total inflation-adjusted Medicare spending in the last 30 days of life, including reimbursements for services provided in inpatient and outpatient settings. The study subjects included U.S. Medicare beneficiaries aged 65 and older who died of breast, prostate, lung, or colorectal cancer between 2011 and 2015. **Results:** Among 59,355 decedents (49.2% female; 21.2% of a non-White race/ethnicity), the factors associated with greater EoL spending were an increased comorbidity burden, the female sex, the Black race, other races/ethnicities, stage III or IV disease, living in a more populated county, and state subsidization of patient Medicare premiums. The EoL spending was lower among older patients; patients living in the Midwest, South, or West; patients living in more rural areas; and patients with a poor performance status. The results were largely consistent across cancer types. **Conclusions:** Our findings can inform targeted intervention development for patients with cancer who are at a higher risk of high-intensity EoL spending, such as decision support tools that facilitate referrals to palliative care for high-risk patients.

## Simple Summary

Medicare patients with cancer account for a significant portion of healthcare spending, particularly in the last month of life. However, the reasons behind this high cost remain unclear. To better understand the factors contributing to these expenses, we analyzed data from older adults who died of breast, prostate, lung, or colorectal cancer. Our study found that higher end-of-life healthcare costs were linked to having more existing health conditions, being female, being Black or of another non-White race, having advanced-stage cancer, living in more populated areas, and receiving state assistance for Medicare premiums. In contrast, lower spending was observed among older patients, those living in rural areas, and those with poorer overall health. These findings suggest that targeted interventions, such as improving access to palliative care for high-risk patients, could help reduce unnecessary medical treatments and improve end-of-life experiences for patients. Understanding these patterns can help policymakers and healthcare providers ensure that resources are used efficiently while prioritizing patient comfort and quality of life.

## 1. Introduction

High-intensity end-of-life (EoL) care for patients with cancer often includes multiple transitions to the hospital and intensive care unit (ICU) and is associated with adverse outcomes, such as declines in patient functional abilities [1,2]. Importantly, aggressive EoL care is often not aligned with patient preferences [3,4] and can result in worse experiences for both caregivers and bereaved family members [4,5]. Intensive medical care, such as the administration of chemotherapy in the last few days of life, generates exorbitant medical spending without reversing the disease course [6,7]. In fact, two previous studies using Medicare data found that the 5–6% of cancer patients who died annually were responsible for about 27–30% of annual Medicare spending, and about 78% of this spending occurred during the last 30 days of life [8,9]. While a portion of this spending represents the intensity of medical needs at the EoL, a significant share is due to the potential overuse of health services that does not align with patient preferences [10]. Given that a small fraction of Medicare beneficiaries is responsible for a significant portion of spending, finding ways to reduce EoL cancer spending provides a unique opportunity to reduce the overall Medicare costs while improving patient centeredness and quality-of-care at the EoL.

There is a large body of research demonstrating that interventions such as upstream palliative care, earlier enrollment in hospice, EoL discussions, and do-not-resuscitate orders are associated with lower medical spending and improved EoL care quality [6,7,11,12]. However, significant disparities exist in the availability and uptake of hospice and palliative care among cancer patients [13,14], which may be partially driven by workforce shortages in these specialties [15,16]. Given these limitations, we cannot universally rely on the current palliative care infrastructure to adequately prevent aggressive EoL care and spending for every single patient with cancer. Therefore, further research is needed to identify which attributes increase a patient’s risk of unnecessary high-intensity care and excessive medical costs at the EoL to help providers focus palliative care interventions toward high-risk patients, and to inform targeted research, education, and policy changes.

Previous research has identified several factors associated with higher healthcare costs among cancer patients, such as age, race/ethnicity, the comorbidity burden, sex, geographic variation, and the disease stage [17,18,19]. However, there has been much less research about which factors contribute to high cancer spending specifically at the EoL. A few recent studies have found that EoL costs are higher for cancer patients who are younger [20,21,22,23], are female [23,24], are members of racial and/or ethnic minorities [23,24], live in urban areas [23], have a higher comorbidity burden [23,24], and live in areas where physicians have less comfort discussing EoL issues [20]. However, the majority of these studies evaluated long spending windows of 6–12 months before death. For Medicare patients with cancer, the EoL spending window is best defined as the last month of life, as previous research has found that almost 80% of spending occurs during the last 30 days of life for this population [9]. Additionally, clinicians often struggle with accurate prognostication, making it difficult to reliably estimate a patient’s final 6–12 months of life. However, the last month of life is a more discernible period for clinicians, providing a clear opportunity for the implementation of targeted interventions. Furthermore, previous studies on this topic have only included one or two types of cancer in their analyses, which greatly limits the generalizability of the findings.

To date, there has been no comprehensive study evaluating the impact of clinical, demographic, socioeconomic, and geographic predictors of high-intensity EoL spending across all common malignancies in the last 30 days of life. Identifying these predictors may help health systems and policymakers determine which patients would benefit most from targeted interventions such as referrals to palliative care, which in turn may reduce the healthcare system’s financial strain caused by high-intensity EoL care. To address these knowledge gaps, we conducted a population-based retrospective cohort study to evaluate and compare the receipt of and predictors for high-intensity EoL spending among Medicare decedents with breast, colorectal, lung, and prostate cancer. Collectively, these four malignancies represent approximately 50% of all new cancer diagnoses in the U.S. [25], enhancing the generalizability of our results as compared to previous research on this topic.

## 2. Methods

### 2.1. Sample Selection and Outcomes

We obtained our patient sample from the SEER-Medicare database, which is a population-based database that combines data from the Surveillance, Epidemiology, and End Results (SEER) program of cancer registries with administrative claims for healthcare services provided to Medicare beneficiaries. Using this dataset, we identified beneficiaries aged 65 years or older diagnosed with the four most common solid tumors, including breast, colorectal, lung, and prostate cancers, who died from cancer between 2011 and 2015. We excluded all the beneficiaries who met any of the following criteria: (1) diagnosed with cancer at autopsy; (2) no histologic confirmation of their cancer diagnosis; (3) unknown dates of cancer diagnosis or death; (4) no continuous coverage for Medicare Parts A/B for 12 months before their death; (5) enrolled in hospice prior to their cancer diagnosis; and (6) no Medicare claims in the 90 days before their death. Our outcome was the total inflation-adjusted Medicare spending in the last 30 days of life, including reimbursements for services provided in inpatient and outpatient settings.

### 2.2. Covariates

All the covariates were selected a priori for inclusion in the statistical models, on the basis of their clinical significance, the existing literature, their relevance for healthcare delivery, and a conceptual framework for the determinants of treatment intensity among seriously ill patients [26]. This selection process was informed through discussions with our team, which comprised practicing oncologists (Wulff-Burchfield, Olszewski, Egan, Trikalinos, and Hugar), palliative care physicians (Wulff-Burchfield), and experts in health services research and EoL healthcare delivery (Bélanger, Baird, and Panagiotou). As a result of this process, we hypothesized that the following categories could influence a patient’s likelihood of experiencing high-intensity EoL spending: patient-level clinical factors, patient-level demographic factors, area-level socioeconomic factors, physician-/practice-related factors, and regional/geographic factors. Consequently, we included as many of these factors in our regression models as our data allowed.

We used the SEER database to obtain patient-specific clinical, tumor, and demographic characteristics recorded at the time of diagnosis, including the patient’s sex, age, marital status, race/ethnicity, and disease stage. We used the Census Tract file to ascertain area-level socioeconomic variables recorded at the time of diagnosis, including the percentage of people living in poverty within the patient’s census tract; whether the patient resided in a tract classified as either all urban, mostly urban, mostly rural, or all rural, as defined by the Census Bureau’s Urban Rural Indicator Code; and the total population of the patient’s county of residence.

Using the Medicare claims billed during the 12 months prior to death, we ascertained each patient’s United States (U.S.) region at death, performance status (approximated by means of a validated claims-based disability indicator and categorized as poor or not poor) [27], comorbidities (measured using the National Cancer Institute (NCI) comorbidity index) [28], and a variable indicating whether the patient’s monthly premium for Part A and/or B coverage was subsidized by the state (“state buy-in”). All the covariates were selected a priori based on clinical knowledge and relevance.

### 2.3. Statistical Analysis

We used univariate linear regression to assess the association of each covariate mentioned above with Medicare spending in the last 30 days of life by computing the regression coefficients and corresponding 95% confidence intervals (CIs). Subsequently, we included all the variables in a multivariable linear regression model, regardless of their statistical significance in the univariate models. In all the models, the outcome variable was a continuous measure of the sum of inpatient and outpatient Medicare spending in the last 30 days of life, and the vector of covariates included those previously listed. We used a Box–Cox transformation [29] for the outcome variable to ensure the normality of the residuals of the linear regression model. This transformation is commonly used to transform non-normal random variables into normal ones and improve the model fit (for further explanation, please see Appendix A).

Our primary analysis pertained to the sample of patients diagnosed with any of the four cancers. We also performed exploratory subgroup analyses for each cancer separately. Finally, we also conducted a descriptive analysis to evaluate whether EoL Medicare spending was higher among patients who received high-intensity EoL care, which we measured through five claims-based quality measures of aggressive EoL care [30,31]. The claims-based indicators of aggressive cancer care at the EoL included death in an acute care hospital, receipt of any oral or parenteral chemotherapy in the last 14 days of life (see Appendix B for more detail), one or more admissions to the intensive care unit (ICU) in the last 30 days of life, two or more emergency department (ER) visits in the last 30 days of life, and two or more inpatient admissions in the last 30 days of life. These indicators have been officially endorsed as quality-of-care measures by the National Quality Forum (NQF) and the American Society of Clinical Oncology (ASCO) and have been widely adopted by researchers studying high-intensity EoL care for patients with cancer [32,33,34]. We used the Statistical Analysis System (SAS) software version 9.4 for data cleaning and management, and we used the Stata software version 18.0 to perform the statistical analyses, in which we set our type I error rate to α = 0.05. This study was designated as exempt by our institution’s Committee for the Protection of Human Subjects (IRB reference #1811002277).

### 2.4. Sensitivity Analysis

Some of the covariates in our analysis (e.g., population in county of residence, marital status, etc.) were documented at the time of the patient’s cancer diagnosis, which may have changed between the patient’s diagnosis and death. For example, a patient may have been living in the Northeastern U.S. when they were diagnosed with cancer, but later moved to the Western U.S. when they received EoL care. To assess the robustness of our findings to these variations, we performed a sensitivity analysis in which we restricted the sample to patients who died within 6 months of their cancer diagnosis, because these covariates are less likely to change during this short time interval.

## 3. Results

### 3.1. Descriptive Results

**Figure 1:**
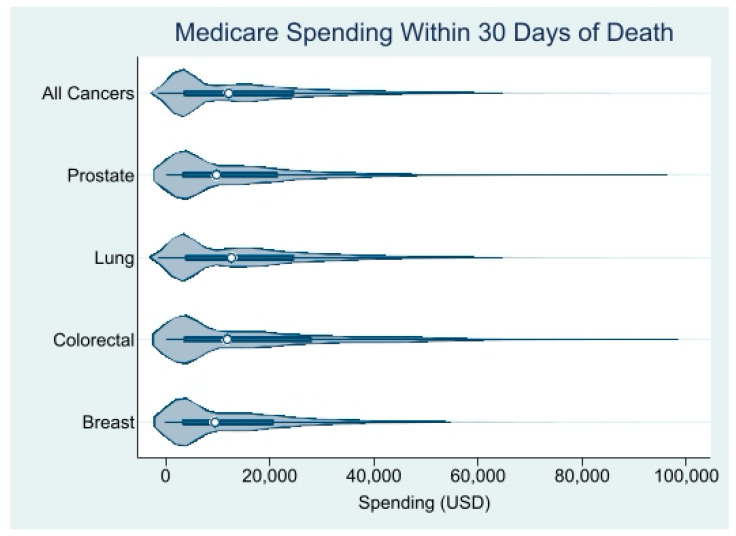
Distribution of unadjusted Medicare spending within 30 days of death. Source: SEER-Medicare dataset.

We identified 59,355 decedents (50.8% males; 78.8% of the White race/ethnicity) who died of lung (*n* = 39,330), colorectal (*n* = 11,806), breast (*n* = 4862), or prostate (*n* = 3357) cancer. The mean age at diagnosis was 76 years and the mean age at death was 77 years. Approximately 54% of the patients had stage IV disease at diagnosis, almost 40% had a poor performance status, and 47% died within 6 months of the diagnosis. The clinical, demographic, socioeconomic, and geographic characteristics are shown in Table 1. Across the four cancers, the median unadjusted Medicare spending in the last 30 days of life was USD 12,325.25 (interquartile range (IQR): USD 3779.10 to USD 25,271.68) (Figure 1). Patients with lung cancer had the highest median EoL Medicare spending, at USD 12,895.34 (IQR: USD 3914.369 to USD 25,246.45), followed closely by patients with colorectal cancer, whose median EoL Medicare spending was USD 12,269.94 (IQR: USD 3633.81 to USD 29,159.15). Patients with prostate cancer had a median EoL Medicare spending of USD 10,071.54 (IQR: USD 3420.203 to USD 21,961.33) and patients with breast cancer had the lowest EoL Medicare spending, with a median of USD 9583.72 (IQR: USD 3327.68 to USD 20,884.07).

**Figure 2:**
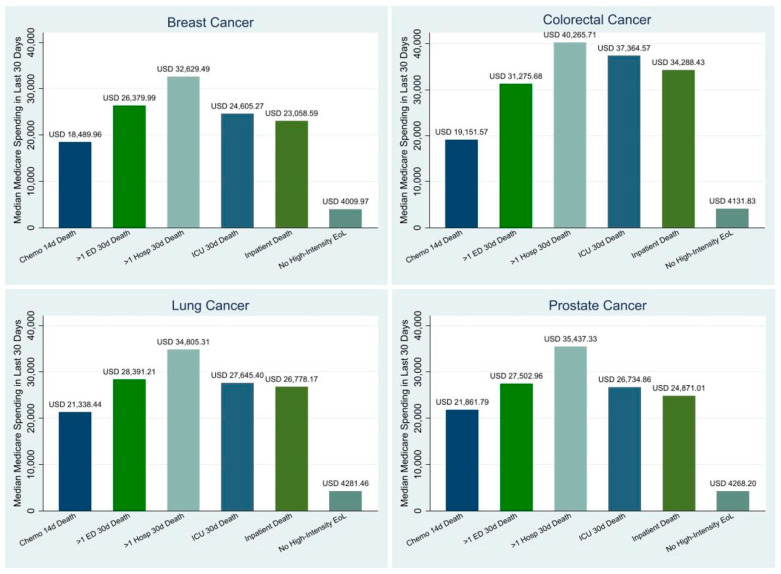
Drivers of Unadjusted Medicare Spending within 30 days of Death by Cancer. **Sources:** SEER-Medicare Dataset.

Figure 2 presents the all-cause unadjusted Medicare spending in the last 30 days of life by both the type of high-intensity EoL care measure and the cancer type. For every single cancer type, having ≥2 inpatient admissions within 30 days of death was associated with the highest median all-cause Medicare spending in the last 30 days of life, ranging from USD 32,629.50 to USD 40,255.70. Conversely, the receipt of any oral or parenteral chemotherapy in the last 14 days of life was associated with the lowest median all-cause Medicare spending in the last 30 days of life for all cancer types, ranging from USD 18,490.00 to USD 21,861.80.

### 3.2. Predictor Results

Table 2 shows the associations of clinical, demographic, socioeconomic, and geographic characteristics with the all-cause Medicare spending in the last 30 days of life. Because we used a Box–Cox transformation of our outcome, the EoL Medicare spending, it was not possible to interpret the coefficients as exact dollar amounts. Rather, positive coefficients indicate that the variable is associated with an increase in EoL Medicare spending, negative coefficients indicate that the variable is associated with a reduction in EoL Medicare spending, and the magnitudes of the coefficients can be used to infer which variables have a more significant impact on the EoL Medicare spending. We found that an increased comorbidity burden (1.06, *p* < 0.001), the female sex (0.33, *p* < 0.001), the Black race (0.91, *p* < 0.001), other races/ethnicities (0.91, *p* < 0.001), stage III at diagnosis (0.37, *p* < 0.01), stage IV at diagnosis (0.39, *p* < 0.001), a higher population in the county of residence (0.56, *p* < 0.001; 1.33, *p* < 0.001), and state buy-in (0.72, *p* < 0.001) were all associated with increased EoL Medicare spending. The EoL Medicare spending was lower for older compared to younger patients (−0.05, *p* < 0.001); patients residing in the Midwest, South, or West compared to the Northeastern U.S. (−1.40, *p* < 0.001; −2.32, *p* < 0.001; −0.66, *p* < 0.001); patients living in all-rural, mostly rural, or mostly urban areas as compared to all-urban areas (−0.62, *p* < 0.001; −0.77, *p* < 0.001; −0.55, *p* < 0.001); and patients with a poor performance status (−5.36, *p* < 0.001).

### 3.3. Regression Results for Additional Analyses

These associations were largely consistent across cancer types except for the level of poverty and the stage at diagnosis. In particular, there was no association between the poverty level and EoL Medicare spending for patients with breast or lung cancer, while patients with prostate cancer residing in a census tract with 5% to <10% poverty, as compared to 0% to <5% poverty, had lower EoL Medicare spending. On the other hand, residing in a census tract with 10% to <20% poverty or 20% to 100% poverty, as compared to 0% to <5% poverty, was associated with higher EoL Medicare spending for patients with colorectal cancer. There were also different associations observed among cancer types for the stage at diagnosis. For all the cancers combined, being diagnosed with stage III or IV cancer, as compared to stage I-II cancer, was associated with more EoL Medicare spending in the last 30 days of life. However, stage III or IV disease was associated with less EoL Medicare spending among patients with colorectal cancer.

The sensitivity analysis included a cohort of 27,821 patients who died within 6 months of their diagnosis and indicated that a higher comorbidity burden, the female sex, the Black race or other races/ethnicities, living in a more populated area, and state buy-in were all associated with more Medicare spending in the last 30 days of life (Table 3). Conversely, an older age; stage III or IV cancer at diagnosis; living in the Midwest, South, or West; living in an all-rural, mostly rural, or mostly urban area; and having a poor performance status were all associated with less Medicare spending in the last 30 days of life.

## 4. Discussion

We conducted a retrospective cohort study of Medicare patients with the four most common cancers to identify predictors of EoL Medicare spending and to examine which types of high-intensity healthcare utilization, as measured through five high-intensity EoL care quality measures, contribute most to EoL Medicare spending. We found that a younger age, the female sex, the Black race/ethnicity, other races/ethnicities, state buy-in, a higher comorbidity burden, a higher stage at diagnosis, a good performance status, and residing in a more populated county, the Northeast, or an all-urban census tract were all associated with greater EoL Medicare spending. Of all the ASCO quality benchmarks, we found that having ≥2 inpatient admissions within 30 days of death was associated with the highest median all-cause EoL Medicare spending. Interestingly, a recent study found that the inpatient hospital costs during the last month of life were 24% lower for cancer decedents who received early palliative care as compared to cancer decedents who did not receive early palliative care [35]. Overall, the median all-cause Medicare spending in the last 30 days of life was USD 20,713.91 higher for patients who received high-intensity EoL care as compared to those who did not, highlighting how high-intensity EoL care places a significant financial strain on the healthcare system.

### 4.1. Demographic Predictors

The association of a younger age with greater EoL Medicare spending is consistent with prior studies [20,21,22,23]. This expenditure difference is largely driven by lower-intensity EoL care among older patients with cancer, which may reflect differences in patient or caregiver preferences by age. Among the patients who were ≤74 at diagnosis, 47.42% received high-intensity EoL care, while only 37.44% of the patients who were ≥85 at diagnosis received high-intensity EoL care. Previous research has suggested that older patients may feel less inclined than younger patients to undergo aggressive treatments only to gain a few more weeks with loved ones [36]. The association of the female sex with greater EoL Medicare spending is also concordant with previous research that assessed cancer spending in the last 6–12 months of life [21,22,23,24].

There is some research demonstrating that, during the EoL, men experience a more rapid de-escalation of care in the ICU setting as compared to female patients [37]. Additionally, because women are more likely to outlive male partners within heterosexual couples, they may rely more on costly long-term care services to fulfill their EoL care needs. These findings could also reflect gender-based differences in EoL care decisions that are made by surrogate decision-makers such as children.

Our finding that the Black race and other minority races are associated with greater EoL Medicare spending as compared to White patients is also consistent with prior research [22,23,24]. Numerous interconnected factors may contribute to racial disparities in EoL spending for cancer patients, including challenges in achieving effective patient–physician communication, limited access to specialized palliative care, a distrust of medical establishments among Black patients stemming from institutional racism, and the influence of faith in shaping preferences for high-intensity EoL care [38,39,40].

### 4.2. Geographic Predictors

Several previous studies have also found that patients with cancer who reside in more populated areas have greater spending in the last year of life [21,22,23]. Higher EoL spending among patients who live in more densely populated areas may signify disparities in the use of EoL healthcare services [41]. Alternatively, these findings may be a reflection of Medicare reimbursing rural providers at lower rates than urban providers for the same services, due to factors such as lower market labor costs. Finally, these results could also indicate that a culture of high-intensity EoL care is more prevalent in urban areas.

### 4.3. Sensitivity Analysis

The predictors of EoL Medicare spending were largely consistent, regardless of how long the patient survived after their cancer diagnosis. However, a more advanced stage at diagnosis was associated with lower EoL Medicare spending among patients who died within 6 months of their diagnosis. Research has found that the accuracy of physician prognostication decreases as the duration of the doctor–patient relationship increases [42,43]. As such, physician prognostication may be more accurate for patients who survive for shorter periods of time after diagnosis, because there is less time for a physician’s judgment to be clouded by their emotional stake in the patient. This improved prognostication may lead patients to forego more highly aggressive care at the EoL, which would decrease EoL spending.

### 4.4. Recommendations for Policy and Practice

Professional societies have attempted to reduce the intensity of EoL care for patients with cancer by establishing EoL care quality metrics and recommending benchmarks for physicians. However, our findings, along with those of other recent studies [44,45,46], indicate that, by themselves, these efforts have not been sufficient to curb excessive and costly care for many patients with cancer who are approaching the EoL. For this reason, moving forward, policymakers should prioritize strategies to more effectively incentivize healthcare providers and health systems to comply with the recommended EoL quality benchmarks. For example, value-based care systems that incorporate financial incentives to enhance the healthcare quality while also lowering costs may offer a viable approach. Notably, the Oncology Care Model (OCM), which is a value-based program managed by the Centers for Medicare and Medicaid Services (CMS), decreased the average total episode expenditures by nearly USD 300 in the first three years of the program [47], primarily through reductions in clinician visits, imaging services, and physician-administered drug use [47,48]. Importantly, this reduced utilization did not have any negative effect on patient survival or quality of life. If the OCM were to incorporate the existing ASCO/NQF high-intensity EoL care quality measures, this model may also be able to reduce unnecessary EoL care utilization and expenditures. Additionally, it may be necessary to invest more in early and continuing education programs that train providers on how to identify which patients are at the greatest risk of high-intensity EoL care, effectively communicate EoL decisions, and successfully refer patients to palliative care and hospice. Further research may also be needed to fully understand all of the factors that may contribute to the avoidance of EoL discussions by both patients and providers.

Our results also indicate that there is an opportunity for the development of decision support tools to alert physicians when they are treating patients at an increased risk of excessive EoL spending. These point-of-care alerts would allow physicians to target EoL interventions, such as serious illness conversations and referrals to palliative care, to those patients who would benefit from them most. Although the decision support intervention from the 1995 SUPPORT study [49] did not precipitate patient outcome improvements, newly developed interventions that leverage recent technology are promising. In fact, a recent study evaluating the impact of an EHR-based intervention that delivered mortality predictions with behavioral nudges to oncology clinicians found that the rate of serious illness conversations was significantly increased among high-mortality-risk patients [50]. This provides support for utilizing the risk factors identified in this study to enhance ongoing machine learning efforts aimed to augment clinician prognostication.

## 5. Limitations

There are some limitations in our study. Firstly, our findings do not reflect the outcomes or experiences of younger patients with these cancers, as the SEER-Medicare dataset includes only patients who are 65 years and older. However, study results obtained from patients with commercial health insurance indicate similar trends in high-intensity EoL care among this population [51]. Second, our EoL spending outcome represents the sum of patient reimbursements for inpatient and outpatient services, and therefore, it does not include costs that are not reimbursed by Medicare. However, while our claims-based outcome may not include the entire sum of EoL healthcare spending, it covers all spending related to potentially unnecessary high-intensity EoL care, which is the focus of this study, and our outcome has been widely used by previous EoL cancer spending studies. Third, the ASCO/NQF high-intensity EoL care benchmarks that are used in the analysis are largely based on research conducted in the early 1990s [30]. However, despite innovations in cancer treatments, the primary features of high-quality EoL medical care have not evolved to include higher-intensity interventions, and therefore, these benchmarks remain appropriate for this analysis.

## 6. Conclusions

In conclusion, Medicare spending in the last 30 days of life was six times higher for patients with indicators of high-intensity EoL care. This demonstrates that high-intensity EoL care not only burdens patients and caregivers, but also adds substantial financial strain to the healthcare system. As such, there is a clear need for policies that incentivize healthcare providers and health systems to better align EoL care intensity with patient preferences and goals of care. We recommend that the risk factors identified in this study be used to inform targeted interventions, such as decision support tools that facilitate referrals to palliative care for high-risk patients.

## Figures and Tables

**Table 1:** Sample characteristics.

	All Cancers (*n* = 59,355)	Breast Cancer (*n* = 4862)	Colorectal Cancer (*n* = 11,806)	Lung Cancer (*n* = 39,330)	Prostate Cancer (*n* = 3357)
Clinical Characteristics					
NCI comorbidity index, mean (SD)	3 (2)	3 (2)	3 (3)	3 (2)	3 (3)
Performance status, No. (%)					
Not poor	35,753 (60.2%)	2449 (50.4%)	6132 (51.9%)	25,022 (63.6%)	2150 (64.0%)
Poor	23,602 (39.8%)	2413 (49.6%)	5674 (48.1%)	14,308 (36.4%)	1207 (36.0%)
Stage at diagnosis, No. (%)					
I–II	10,182 (17.2%)	1540 (31.7%)	2554 (21.6%)	5173 (13.2%)	915 (27.3%)
III	13,391 (22.6%)	920 (18.9%)	2649 (22.4%)	9722 (24.7%)	100 (3.0%)
IV	31,874 (53.7%)	1825 (37.5%)	5495 (46.5%)	22,614 (57.5%)	1940 (57.8%)
Death within 6 months of diagnosis, No. (%)					
No	31,534 (53.1%)	3595 (73.9%)	6677 (56.6%)	18,512 (47.1%)	2750 (81.9%)
Yes	27,821 (46.9%)	1267 (26.1%)	5129 (43.4%)	20,818 (52.9%)	607 (18.1%)
Demographic Characteristics					
Age at diagnosis, mean years (SD)	76 (8)	77 (9)	79 (8)	76 (7)	77 (8)
Age at death, mean years (SD)	77 (8)	79 (9)	80 (8)	76 (7)	79 (8)
Year of death, No. (%)					
2011	5994 (10.1%)	311 (6.4%)	1187 (10.1%)	4318 (11.0%)	178 (5.3%)
2012	11,099 (18.7%)	679 (14.0%)	2078 (17.6%)	7859 (20.0%)	483 (14.4%)
2013	13,267 (22.4%)	1044 (21.5%)	2597 (22.0%)	8933 (22.7%)	693 (20.6%)
2014	14,130 (23.8%)	1286 (26.5%)	2883 (24.4%)	9016 (22.9%)	945 (28.2%)
2015	14,865 (25.0%)	1542 (31.7%)	3061 (25.9%)	9204 (23.4%)	1058 (31.5%)
Sex, No. (%)					
Female	29,187 (49.2%)	4809 (98.9%)	6209 (52.6%)	18,169 (46.2%)	0.00 (0.0%)
Male	30,168 (50.8%)	53 (1.1%)	5597 (47.4%)	21,161 (53.8%)	3357 (100.0%)
Race/ethnicity, No. (%)					
White	46,765 (78.8%)	3758 (77.3%)	8844 (74.9%)	31,665 (80.5%)	2498 (74.4%)
Black	5863 (9.9%)	616 (12.7%)	1328 (11.2%)	3453 (8.8%)	466 (13.9%)
Hispanic	3261 (5.5%)	274 (5.6%)	855 (7.2%)	1892 (4.8%)	240 (7.1%)
Other ^	3466 (5.8%)	214 (4.4%)	779 (6.6%)	2320 (5.9%)	153 (4.6%)
Marital status, No. (%)					
Married	27,345 (46.1%)	1556 (32.0%)	4851 (41.1%)	19,123 (48.6%)	1815 (54.1%)
Not married	28,942 (48.8%)	2957 (60.8%)	6340 (53.7%)	18,507 (47.1%)	1138 (33.9%)
Geographic/Socioeconomic Characteristics					
U.S. region at death, No. (%)					
Northeast	10,936 (18.4%)	968 (19.9%)	2297 (19.5%)	7056 (17.9%)	615 (18.3%)
Midwest	7337 (12.4%)	606 (12.5%)	1436 (12.2%)	4898 (12.5%)	397 (11.8%)
South	17,370 (29.3%)	1349 (27.7%)	3113 (26.4%)	12,050 (30.6%)	858 (25.6%)
West	23,679 (39.9%)	1936 (39.8%)	4951 (41.9%)	15,306 (38.9%)	1486 (44.3%)
Population in county of residence, No. (%)					
249,999 or less	16,477 (27.8%)	1231 (25.3%)	3136 (26.6%)	11,195 (28.5%)	915 (27.3%)
250,000 to 999,999	12,507 (21.1%)	1014 (20.9%)	2473 (20.9%)	8298 (21.1%)	722 (21.5%)
1,000,000 or more	30,318 (51.1%)	2613 (53.7%)	6183 (52.4%)	19,804 (50.4%)	1718 (51.2%)
Rural/urban area at diagnosis, No. (%)					
All rural	5425 (9.1%)	374 (7.7%)	993 (8.4%)	3761 (9.6%)	297 (8.8%)
All urban	34,935 (58.9%)	2917 (60.0%)	7194 (60.9%)	22,907 (58.2%)	1917 (57.1%)
Mostly rural	5100 (8.6%)	348 (7.2%)	889 (7.5%)	3564 (9.1%)	299 (8.9%)
Mostly urban	13,058 (22.0%)	1,021 (21.0%)	2590 (21.9%)	8731 (22.2%)	716 (21.3%)
Poverty, No. (%)					
0% to <5% poverty	10,204 (17.2%)	918 (18.9%)	2032 (17.2%)	6634 (16.9%)	620 (18.5%)
5% to <10% poverty	14,420 (24.3%)	1234 (25.4%)	2856 (24.2%)	9497 (24.1%)	833 (24.8%)
10% to <20% poverty	18,223 (30.7%)	1371 (28.2%)	3625 (30.7%)	12,237 (31.1%)	990 (29.5%)
20% to 100% poverty	15,092 (25.4%)	1243 (25.6%)	3048 (25.8%)	9943 (25.3%)	858 (25.6%)
State buy-in, No. (%)					
No	45,838 (77.2%)	3640 (74.9%)	8748 (74.1%)	30,745 (78.2%)	2705 (80.6%)
Yes	13,517 (22.8%)	1222 (25.1%)	3058 (25.9%)	8585 (21.8%)	652 (19.4%)

Sources: SEER-Medicare dataset, Census Tract file. ^ Cell sizes were too small to show races within the “Other” category.

**Table 2:** Multivariable regression results: Medicare spending in the last 30 days of life (full sample).

	All Cancers	Breast	Colorectal	Lung	Prostate
	Coefficient (95% CI)	Coefficient (95% CI)	Coefficient (95% CI)	Coefficient (95% CI)	Coefficient (95% CI)
Clinical Predictors					
NCI Comorbidity Index	1.06 *** (1.03 to 1.09)	0.99 *** (0.87 to 1.10)	1.07 *** (0.99 to 1.15)	1.10 *** (1.06 to 1.14)	0.94 *** (0.80 to 1.08)
Performance Status					
Not poor	0 [Reference]	0 [Reference]	0 [Reference]	0 [Reference]	0 [Reference]
Poor	−5.36 *** (−5.53 to −5.20)	−4.62 *** (−5.18 to −4.06)	−6.31 *** (−6.71 to −5.92)	−5.33 *** (−5.52 to −5.13)	−4.43 *** (−5.17 to −3.68)
Stage at Diagnosis					
I–II	0 [Reference]	0 [Reference]	0 [Reference]	0 [Reference]	0 [Reference]
III	0.37 ** (0.13 to 0.61)	0.52 (−0.20 to 1.25)	−0.83 ** (−1.38 to −0.29)	0.89 *,** (0.58 to 1.19)	0.82 (−1.21 to 2.85)
IV	0.39 *** (0.18 to 0.60)	1.62 *** (1.01 to 2.23)	−1.72 *** (−2.20 to −1.24)	1.09 *** (0.81 to 1.37)	1.04 ** (0.26 to 1.81)
Demographic Predictors					
Age at Diagnosis	−0.05 *** (−0.06 to −0.04)	−0.16 *** (−0.19 to −0.13)	−0.04 ** (−0.06 to −0.01)	−0.06 *** (−0.07 to −0.05)	−0.08 *** (−0.12 to −0.03)
Sex					
Male	0 [Reference]	0 [Reference]	0 [Reference]	0 [Reference]	
Female	0.33 *** (0.16 to 0.49)	1.97 (−0.69 to 4.62)	0.18 (−0.22 to 0.58)	0.34 *** (0.15 to 0.54)	
Race/Ethnicity					
White	0 [Reference]	0 [Reference]	0 [Reference]	0 [Reference]	0 [Reference]
Black	0.91 *** (0.63 to 1.19)	1.72 *** (0.83 to 2.61)	0.50 (−0.15 to 1.15)	0.87 *** (0.52 to 1.21)	0.80 (−0.36 to 1.96)
Hispanic	0.03 (−0.33 to 0.38)	−0.33 (−1.53 to 0.88)	−0.46 (−1.23 to 0.31)	0.18 (−0.26 to 0.63)	−0.66 (−2.11 to 0.79)
Other ^	0.91 *** (0.56 to 1.27)	−0.17 (−1.55 to 1.22)	0.13 (−0.68 to 0.95)	1.20 *** (0.78 to 1.62)	1.07 (−0.69 to 2.82)
Marital Status					
Married	0 [Reference]	0 [Reference]	0 [Reference]	0 [Reference]	0 [Reference]
Not married	0.16 (−0.01 to 0.33)	−0.33 (−0.92 to 0.27)	0.14 (−0.27 to 0.55)	0.20 * (0.00 to 0.40)	0.15 (−0.60 to 0.89)
Geographic/Socioeconomic Predictors					
U.S. Region at Death					
Northeast	0 [Reference]	0 [Reference]	0 [Reference]	0 [Reference]	0 [Reference]
Midwest	−1.40 *** (−1.69 to −1.11)	−1.25 ** (−2.22 to −0.27)	−1.62 *** (−2.33 to −0.92)	−1.36 *** (−1.70 to −1.01)	−1.18 (−2.5 to 0.14)
South	−2.32 *** (−2.59 to −2.06)	−2.16 *** (−3.06 to −1.27)	−2.30 *** (−2.94 to −1.66)	−2.26 *** (−2.57 to −1.95)	−2.53 *** (−3.74 to −1.32)
West	−0.66 *** (−0.89 to −0.43)	−0.37 (−1.14 to 0.41)	−0.74 ** (−1.28 to −0.19)	−0.67 *** (−0.94 to −0.40)	−0.21 (−1.23 to 0.82)
Population in County of Residence					
249,999 or less	0 [Reference]	0 [Reference]	0 [Reference]	0 [Reference]	0 [Reference]
250,000–999,999	0.56 *** (0.32 to 0.81)	0.83 (−0.03 to 1.70)	1.07 *** (0.46 to 1.68)	0.46 ** (0.17 to 0.75)	−0.18 (−1.29 to 0.93)
1,000,000 or more	1.33 *** (1.10 to 1.57)	1.91 *** (1.09 to 2.73)	1.44 *** (0.86 to 2.03)	1.23 *** (0.95 to 1.51)	1.17 * (0.11 to 2.24)
Rural/Urban Area at Diagnosis					
All urban	0 [Reference]	0 [Reference]	0 [Reference]	0 [Reference]	0 [Reference]
All rural	−0.62 *** (−0.95 to −0.30)	0.09 (−1.09 to 1.27)	−0.65 (−1.45 to 0.15)	−0.61 *** (−0.98 to −0.23)	−1.97 ** (−3.4 to −0.53)
Mostly rural	−0.77 *** (−1.08 to −0.46)	0.79 (−0.33 to 1.91)	−0.65 (−1.45 to 0.15)	−0.93 *** (-1.29 to -0.57)	−0.87 (−2.22 to 0.48)
Mostly urban	−0.55 *** (−0.76 to −0.34)	−0.43 (−1.15 to 0.29)	−0.39 (−0.91 to 0.13)	−0.59 *** (−0.84 to −0.34)	−1.15 * (−2.11 to −0.19)
Poverty					
0–<5% poverty	0 [Reference]	0 [Reference]	0 [Reference]	0 [Reference]	0 [Reference]
5–<10% poverty	−0.10 (−0.35 to 0.14)	−0.27 (−1.08 to 0.53)	0.16 (−0.44 to 0.76)	−0.11 (−0.40 to 0.18)	−1.22 * (−2.3 to −0.15)
10–<20% poverty	0.07 (−0.18 to 0.31)	0.48 (−0.35 to 1.31)	0.76 * (0.16 to 1.35)	−0.19 (−0.48 to 0.10)	−0.45 (−1.55 to 0.65)
20–100% poverty	0.09 (−0.18 to 0.36)	−0.03 (−0.94 to 0.89)	1.05 ** (0.40 to 1.70)	−0.14 (−0.46 to 0.18)	−0.90 (−2.09 to 0.3)
State Buy-In					
No	0 [Reference]	0 [Reference]	0 [Reference]	0 [Reference]	0 [Reference]
Yes	0.72 *** (0.52 to 0.93)	0.31 (−0.38 to 1.00)	0.18 (−0.30 to 0.66)	0.86 *** (0.61 to 1.10)	1.19 * (0.2 to 2.18)

Sources: SEER-Medicare dataset, Census Tract file. ^ Cell sizes were too small to show races within the “Other” category. Notes: * *p*-value < 0.05; ** *p*-value < 0.01; *** *p*-value < 0.001.

**Table 3:** Multivariable regression results: Medicare spending in the last 30 days of life (sensitivity sample).

	All Cancers	Breast	Colorectal	Lung	Prostate
	Coefficient (95% CI)	Coefficient (95% CI)	Coefficient (95% CI)	Coefficient (95% CI)	Coefficient (95% CI)
Clinical Predictors					
NCI Comorbidity Index	0.94 *** (0.89 to 0.98)	0.90 *** (0.69 to 1.11)	0.82 *** (0.71 to 0.93)	1.01 *** (0.96 to 1.07)	0.79 *** (0.49 to 1.09)
Performance Status					
Not poor	0 [Reference]	0 [Reference]	0 [Reference]	0 [Reference]	0 [Reference]
Poor	−4.98 *** (−5.24 to −4.73)	−3.83 *** (−4.89 to −2.76)	−5.81 *** (−6.41 to −5.20)	−5.00 *** (−5.29 to −4.72)	−4.39 *** (−6.08 to −2.69)
Stage at Diagnosis					
I–II	0 [Reference]	0 [Reference]	0 [Reference]	0 [Reference]	0 [Reference]
III	−1.35 *** (−1.78 to −0.92)	0.81 (−1.09 to 2.70)	−0.55 (−1.41 to 0.32)	−0.25 (−0.80 to 0.29)	7.53 (−2.20 to 17.26)
IV	−2.24 *** (−2.62 to −1.86)	0.86 (−0.58 to 2.30)	−4.71 *** (−5.43 to −4.00)	−0.60 * (−1.10 to −0.10)	4.02 *** (1.90 to 6.14)
Demographic Predictors					
Age at Diagnosis	−0.08 *** (−0.10 to −0.06)	−0.20 *** (−0.26 to −0.13)	−0.05 ** (−0.09 to −0.02)	−0.12 *** (−0.14 to −0.10)	−0.04 (−0.14 to 0.06)
Sex					
Male	0 [Reference]	0 [Reference]	0 [Reference]	0 [Reference]	
Female	0.49 *** (0.26 to 0.72)	1.44 (−3.87 to 6.76)	−0.12 (−0.71 to 0.46)	0.59 *** (0.33 to 0.85)	
Race/Ethnicity					
White	0 [Reference]	0 [Reference]	0 [Reference]	0 [Reference]	0 [Reference]
Black	0.66 ** (0.26 to 1.07)	0.83 (−0.79 to 2.45)	0.23 (−0.74 to 1.20)	0.66 ** (0.19 to 1.12)	0.87 (−1.57 to 3.31)
Hispanic	−0.12 (−0.64 to 0.40)	−1.89 (−4.28 to 0.50)	−1.37 * (−2.55 to −0.19)	0.27 (−0.33 to 0.86)	−1.07 (−4.36 to 2.22)
Other ^	0.72 ** (0.20 to 1.23)	−1.28 (−4.00 to 1.44)	0.27 (−0.98 to 1.52)	0.97 *** (0.40 to 1.54)	2.67 (−2.85 to 8.19)
Marital Status					
Married	0 [Reference]	0 [Reference]	0 [Reference]	0 [Reference]	0 [Reference]
Not married	0.08 (−0.16 to 0.32)	0.42 (−0.70 to 1.55)	0.06 (−0.54 to 0.66)	0.01 (−0.26 to 0.27)	−0.25 (−1.83 to 1.34)
Geographic/Socioeconomic Predictors					
U.S. Region at Death					
Northeast	0 [Reference]	0 [Reference]	0 [Reference]	0 [Reference]	0 [Reference]
Midwest	−1.66 *** (−2.07 to −1.24)	−2.08 * (−3.81 to −0.35)	−1.58 ** (−2.60 to −0.57)	−1.65 *** (−2.12 to −1.18)	−2.36 (−5.37 to 0.65)
South	−2.83 *** (−3.21 to −2.46)	−3.71 *** (−5.29 to −2.12)	−2.27 *** (−3.21 to −1.34)	−2.72 *** (−3.15 to −2.30)	−5.02 *** (−7.71 to −2.34)
West	−0.92 *** (−1.25 to −0.59)	−1.26 (−2.66 to 0.14)	−0.97 * (−1.77 to −0.17)	−0.85 *** (−1.22 to −0.47)	−1.50 (−3.81 to 0.82)
Population in County of Residence					
249,999 or less	0 [Reference]	0 [Reference]	0 [Reference]	0 [Reference]	0 [Reference]
250,000–999,999	0.42 * (0.07 to 0.77)	1.19 (−0.42 to 2.80)	0.60 (−0.28 to 1.48)	0.36 (−0.03 to 0.75)	−0.10 (−2.53 to 2.34)
1,000,000 or more	1.25 *** (0.91 to 1.58)	1.67 * (0.15 to 3.19)	1.34 ** (0.49 to 2.20)	1.22 *** (0.85 to 1.60)	1.31 (−0.99 to 3.61)
Rural/Urban Area at Diagnosis					
All urban	0 [Reference]	0 [Reference]	0 [Reference]	0 [Reference]	0 [Reference]
All rural	−0.60 * (−1.05 to −0.14)	1.00 (−1.16 to 3.16)	−1.20 * (−2.35 to −0.04)	−0.58 * (−1.09 to −0.07)	−2.05 (−5.51 to 1.41)
Mostly rural	−0.79 ** (−1.23 to −0.34)	1.24 (−0.83 to 3.31)	−1.24 * (−2.41 to −0.08)	−0.76 ** (−1.26 to −0.27)	−0.68 (−3.90 to 2.55)
Mostly urban	−0.48 ** (−0.79 to −0.18)	−0.17 (−1.53 to 1.19)	−0.85 * (−1.60 to −0.09)	−0.49 ** (−0.83 to −0.15)	−0.61 (−2.61 to 1.38)
Poverty					
0–<5% poverty	0 [Reference]	0 [Reference]	0 [Reference]	0 [Reference]	0 [Reference]
5–<10% poverty	−0.02 (−0.38 to 0.34)	−0.62 (−2.11 to 0.86)	0.09 (−0.79 to 0.97)	0.02 (−0.38 to 0.42)	0.23 (−2.19 to 2.65)
10–<20% poverty	0.08 (−0.28 to 0.43)	−0.50 (−2.03 to 1.03)	0.84 (−0.04 to 1.71)	−0.12 (−0.52 to 0.28)	1.29 (−1.31 to 3.89)
20–100% poverty	0.12 (−0.27 to 0.51)	0.34 (−1.40 to 2.07)	0.76 (−0.19 to 1.72)	−0.10 (−0.54 to 0.34)	1.73 (−0.92 to 4.39)
State Buy-In					
No	0 [Reference]	0 [Reference]	0 [Reference]	0 [Reference]	0 [Reference]
Yes	0.75 *** (0.46 to 1.04)	0.02 (−1.27 to 1.31)	0.54 (−0.17 to 1.25)	0.83 *** (0.50 to 1.16)	0.00 (−2.19 to 2.20)

Sources: SEER-Medicare dataset, Census Tract file. ^ Cell sizes were too small to show races within the “Other” category. Notes: * *p*-value < 0.05; ** *p*-value < 0.01; *** *p*-value < 0.001.

## Data Availability

No data are available from the authors. SEER-Medicare data can be requested from the National Cancer Institute: https://healthcaredelivery.cancer.gov/seermedicare/ (accessed on 24 February 2025).

## Appendix A

### Model Fit Based on Box–Cox Transformation

Even though Box–Cox-transforming the outcome variable before using it in the model does not necessarily translate to normally distributed residuals, the transformation resulted in an improved model fit based on the Akaike information criterion (AIC); lower AIC values indicate a better-fitting model. The AIC of the model when using the Box–Cox-transformed outcome was equal to 350,878.40, while the AIC was equal to 1,113,823.00 when using the raw (untransformed) cost outcome.

## Appendix B

### Chemotherapy Endpoint

The endpoint of the “use of chemotherapy within the last 14 days of life” was examined in a subgroup of patients who had available Medicare Part A and B (to account for office-administered chemotherapy) and Part D (to account for orally administered prescriptions for antineoplastic agents) claims, with a minimum of 3 months of coverage under Part D within the last 6 months of life. Chemotherapy drugs were identified using the Healthcare Common Procedure Coding System (HCPCS) billing codes for outpatient hospital or physician office administration (Cancer Medications Enquiry Database (CanMED), the Surveillance Research Program SEER website tool, version 1.12.2: Division of Cancer Control and Population Sciences, National Cancer Institute; available from https://seer.cancer.gov/oncologytoolbox/canmed/, accessed on 12 February 2025) and the National Cancer Institute database of oral anti-cancer drugs for prescription drugs (A to Z List of Cancer Drugs: National Cancer Institute; available from https://www.cancer.gov/about-cancer/treatment/drugs, accessed on 12 February 2025).
